# Albuminuria Predicts a Rapid Decline in Kidney Function in 2 International, Longitudinal Cohorts of Adults With Sickle Cell Anemia

**DOI:** 10.1002/ajh.70155

**Published:** 2025-12-03

**Authors:** Pablo Bartolucci, Étienne Audureau, Vincent Audard, Frédéric Galactéros, Guillaume Lettre, Charmaine Anthony, Olufolake Egbujo, Jin Han, Maria Armila Ruiz, James P. Lash, Victor R. Gordeuk, Xu Zhang, Santosh L. Saraf

**Affiliations:** ^1^ Hôpital Henri Mondor Assistance Publique des Hôpitaux de Paris (AP‐HP) Univ Paris Est‐Créteil Créteil France; ^2^ Nephrology and Transplantation Department, Henri Mondor University Hospital, APHP Institut Mondor de Recherche Biomédicale (IMRB), Univ Paris Est‐Créteil, Institut National de la Santé et de la Recherche Médicale (INSERM) U955 Créteil France; ^3^ Red Cell Genetic Disease Unit, Hôpital Henri‐Mondor, AP‐HP Université Paris Est Créteil France; ^4^ Department of Medicine Université de Montréal Montreal Québec Canada; ^5^ Montreal Heart Institute Montreal Quebec Canada; ^6^ Novartis Global Health East Hanover New Jersey USA; ^7^ Department of Pharmacy Practice University of Illinois Chicago Chicago Illinois USA; ^8^ Division of Hematology & Oncology, Department of Medicine University of Illinois Chicago Chicago Illinois USA; ^9^ Division of Nephrology, Department of Medicine University of Illinois Chicago Chicago Illinois USA

**Keywords:** albuminuria, kidney, sickle cell disease, tobacco smoking

## Abstract

Chronic kidney disease (CKD) is common and a major contributor to increased morbidity and early mortality in people with sickle cell anemia (SCA). Urine albumin‐to‐creatinine ratio (uACR) is recommended to identify patients with SCA‐related CKD but its utility in predicting long‐term kidney dysfunction remains unclear in this patient population. In two independent, longitudinal cohorts of patients with hemoglobin SS or Sβ^0^‐thalassemia (USA: *n* = 268, median follow‐up 6 years; France: *n* = 310, median follow‐up 8.2 years) we investigated the utility of uACR, as well as other clinical and modifiable risk factors, for predicting a decline in kidney function as determined by the rate of estimated glomerular filtration rate (eGFR) decline. Using linear mixed‐effects models, a higher baseline uACR independently predicted a faster rate of eGFR decline as well as a more rapid annual eGFR decline, defined as ≥ 3 mL/min/1.73m^2^ (*p* ≤ 0.009). Furthermore, baseline uACR of ≥ 100 mg/g creatinine was independently associated with the rate of eGFR decline and rapid annual eGFR decline in both cohorts. Tobacco smoking was also associated with a faster rate of eGFR decline and was congruous between the two cohorts. In conclusion, we demonstrate that uACR is an important clinical tool that predicts a more rapid decline in kidney function and should be routinely monitored in people with SCA. Our data also support preventative care to reduce tobacco smoking for mitigating the risk of CKD progression in this high‐risk population.

## 1 Introduction

Sickle cell disease is an inherited red blood cell disorder affecting approximately 8 million people worldwide [1]. With advances in therapy, there has been a paradigm shift for causes of early mortality from acute infectious and vaso‐occlusive complications to chronic organ damage, which now represents the cause of death in approximately 50% of adults with sickle cell anemia (SCA) [2]. Chronic kidney disease (CKD) is observed in up to half of adults with SCA and contributes to approximately 18% of the mortality [3, 4].

In SCA, the estimated glomerular filtration rate (eGFR) typically follows a pattern of being in the hyperfiltration range in childhood, followed by a plateau between the ages of 10 and 20 years old, and then a rapid decline in some patients in adulthood [5]. There is substantial variability in which patients follow this pattern of eGFR change, emphasizing the need to identify SCA patients at high risk for rapid eGFR decline. The mean annual rate of eGFR decline in adults with SCA ranges between 2.05 and 2.53 mL/min/1.73m^2^ per year [6, 7, 8, 9]. This rate of eGFR decline has been consistently more rapid than what has been observed in those with sickle cell trait (1.67 mL/min/1.73m^2^ per year) and in healthy control populations (1.22 to 1.27 mL/min/1.73m^2^ per year) [6, 7]. Furthermore, a rapid decline in eGFR of > 3 mL/min/1.73m^2^ per year is observed in a third of adults with SCA [6, 9, 10] and predicts a 2‐fold greater risk of CKD progression, defined in that study by an eGFR of < 90 mL/min/1.73m^2^ and a 50% decline in eGFR from baseline, and a 2.4‐fold greater risk of early mortality [9].

The urine albumin‐to‐creatinine ratio (uACR) is an early manifestation of kidney damage that may identify patients at high risk of CKD progression. In patients with type 2 diabetes, increased uACR predicts a 4.4‐fold greater risk of CKD progression, defined by a doubling of serum creatinine or requiring renal replacement therapy support [11]. Although an elevated uACR is common in SCA patients, observed in up to 27% of children and 68% of adults with SCA, [5] and screening uACR is recommended by the American Society of Hematology 2019 guidelines starting at age 10 years, [12] there is a dearth of data demonstrating that this screening tool identifies SCA patients at high risk for a rapid decline in kidney dysfunction. Furthermore, the predictive role of albuminuria to predict a rapid decline in kidney function has been inconsistent and the association of genetic risk factors or modifiable risk factors with a rapid eGFR decline remains unclear. It is imperative that we identify SCA individuals at high risk for a rapid eGFR decline in order to guide strategies for closer monitoring of kidney function, avoidance of nephrotoxic agents (e.g., non‐steroidal anti‐inflammatory drugs and certain antibiotics), and early institution of SCA‐disease modifiers (e.g., hydroxyurea, curative or transformative therapies) or reno‐protective therapies (e.g., renal‐angiotensin‐aldosterone system [RAAS] inhibitors).

To address these gaps in our knowledge, we evaluated the utility of the uACR as well as genetic, clinical, and laboratory risk factors associated with eGFR decline in a longitudinal registry of adults with hemoglobin (Hb) SS or Sβ^0^‐thalassemia sickle cell disease from the United States and repeated our analyses in a separate cohort of adults with Hb SS or Sβ^0^‐thalassemia sickle cell disease from France. These independent, international cohorts allow us to determine whether uACR can predict longitudinal kidney function outcomes in different clinical and environmental settings.

## 2 Methods

The study was approved by the Institutional Review Boards of the participating institutions and the subjects provided written informed consent in accordance with the Declaration of Helsinki. The University of Illinois Chicago (UIC) cohort included 268 Hb SS or Sβ^0^‐thalassemia patients recruited into a registry between August 2010 and March 2016 with at least 3 months of longitudinal eGFR data. Clinical data were obtained from the electronic medical charts longitudinally from the time of enrolment. The Henri Mondor cohort included 310 Hb SS or Sβ^0^‐thalassemia patients from the GEN‐MOD cohort recruited from 2003 to 2015 with at least 3 months of longitudinal eGFR data. The GEN‐MOD cohort recruited patients prior to starting hydroxyurea as well as any siblings with Hb SS or Sβ^0^‐thalassemia genotypes, with the latter representing those on hydroxyurea in this cohort's analysis. Data were prospectively collected at inclusion.

The eGFR was calculated using the Chronic Kidney Disease Epidemiology Collaboration (CKD‐EPI) equation (2021), which did not include the race coefficient [13]. All follow‐up outpatient eGFR values (UIC: *n* = 4764 total values; Henri Mondor: 5656) were included in the eGFR slope analysis. We applied a conversion factor of 0.113 to convert the uACR in the Henri Mondor cohort (SI units of mg/mmol) to U.S. units (mg/g).

The *APOL1* G1 and G2 genotyping were performed as previously described by polymerase chain reaction (PCR) [14, 15]. *APOL1* high risk was defined as homozygous or compound heterozygosity for the G1 and G2 (G1/G1, G2/G2 or G1/G2) risk alleles. The a priori uACR thresholds of ≥ 30 and ≥ 300 mg/g were determined using the Kidney Disease Improving Global Outcomes (KDIGO) albuminuria thresholds [16]. The UACR threshold of ≥ 100 mg/g was also chosen a priori based on this degree of albuminuria being associated with the persistence of albuminuria in SCA [10, 17]. A vaso‐occlusive episode was defined in both cohorts as presentation to the emergency department or hospitalization for an acute pain episode.

## 3 Statistical Analysis

The association of clinical variables with eGFR decline was assessed using a linear mixed effects model by restricted maximum likelihood. Levels of eGFR were regressed by uACR or uACR categories, time (years starting from baseline), and uACR by time interaction, with random intercepts and random slopes. Covariates were first individually tested for time‐interaction effects on eGFR. Covariates with *p* < 0.1 in individual tests were then simultaneously tested for time‐interaction effects on eGFR and selected using backward elimination at *p* = 0.1. To capture variance due to non‐linear trends, time was further modeled by natural cubic splines of up to six degrees of freedom. Models with time splines as fixed effects, random effects or both were compared using maximum likelihood estimation and Bayesian information criterion (BIC). The final model included fixed linear time and random time splines of two degrees of freedom for the UIC cohort (Δ_BIC_ = −144 compared to random time slopes) and five degrees of freedom for the Henri Mondor cohort (Δ_BIC_ = −142 compared to random time slopes). Subjects with missing variables were not included in the multivariable models. LDH and uACR were log‐transformed in the analyses.

Rapid eGFR decline, defined as eGFR decreasing by more than 3 mL/min/1.73m^2^ per year, was regressed by uACR or uACR categories using a logistic regression model. Covariates were first individually tested for the main effect on rapid eGFR decline. Covariates with *p* < 0.1 in individual tests were then simultaneously tested for main effects on eGFR decline and selected using backward elimination at *p* = 0.1. Analyses were conducted using R version 4.03.

## 4 Results

### 4.1 Baseline Characteristics

Baseline characteristics of the UIC cohort included a median age of 32 years, 57% female, 50% on hydroxyurea, 20% on RAAS‐inhibitor therapy, and 24% tobacco smokers (Table 1). High‐risk *APOL1* status was observed in 11.5% of the cohort.

**TABLE 1:** Baseline characteristics of the adult hemoglobin SS or Sβ^0^‐thalassemia cohorts.

Variable	UIC *N* = 268	Henri Mondor *N* = 310
Age (years)	32 (25–43)	33 (27–40)
Female sex (%)	154 (57%)	173 (56%)
Diabetes mellitus (%)	29 (11%)	1 (0.3%)
Hydroxyurea use (%)	133 (50%)	28 (9%)
RAAS inhibitors use (%)	53 (20%)	1 (0.3%)
Chronic RBC transfusions (%)	26 (10%)	NA^a^
Tobacco smoking use (%)	64 (24%)	23 (7%)
Body mass index (kg/m^2^)	23 (21–26)	22 (19–24)
Systolic blood pressure (mmHg)	117 (110–128)	115 (108–125)
WBC count (× 10^3^/μL)	9.4 (7.4–11.9)	10.3 (8.4–12.2)
Hemoglobin (g/dL)	8.7 (7.8–9.5)	8.7 (7.9–9.5)
Platelet count (× 10^3^/μL)	411 (314–509)	353 (275–447)
Hemoglobin F (%)	5.6 (2.6–11.8)	5.9 (2.9–9.2)
Absolute retic (× 10^3^/μL)	305 (206–415)	224 (174–287)
LDH (μ/L)	372 (280–482)	395 (319–509)
VOE Frequency (per year)	2.0 (0.5–4.0)	0.3 (0.1–0.6)
uACR (mg/g)	40 (13–205)	33 (9–132)
eGFR (mL/min/1.73m^2^)	122 (101–131)	118 (106–129)
*APOL1* high‐risk (%)	27/235 (11.5%)	22/286 (8%)

Abbreviations: LDH, lactate dehydrogenase; RAAS, renin‐angiotensin‐aldosterone system; RBC, red blood cell; WBC, white blood cell; uACR, urine albumin‐to‐creatinine ratio; VOE, vaso‐occlusive episodes.

^a^ Chronic RBC transfusions were an exclusion criterion for entry in the Henri Mondor registry cohort.

Baseline characteristics of the Henri Mondor cohort at the first eGFR examination included a median age of 33 years, 56% female, 9% on hydroxyurea therapy, 0.3% on RAAS‐inhibitor therapy, and 7% tobacco smokers (Table 1). High‐risk *APOL1* status was observed in 8% of the cohort.

### 4.2 Rate of eGFR Decline

In the UIC cohort, the median follow‐up was 6.0 years (interquartile range, 3.3–8.2 years) and the mean rate of eGFR decline was −2.14 mL/min/1.73m^2^ per year. On univariate analysis, baseline variables associated with a more rapid annual eGFR decline included older age, diabetes mellitus, the use of RAAS inhibitors, tobacco smoking, higher systolic blood pressure, higher uACR, and coinheritance of high‐risk *APOL1* status while a higher hemoglobin concentration was associated with a less rapid annual eGFR decline (Table 2).

**TABLE 2:** Baseline variables associated with estimated glomerular filtration rate.

Variable	UIC		Henri Mondor	
	*β* ± standard error	*p*	*β* ± standard error	*p*
Age (years)	−0.041 ± 0.02	0.041	−0.064 ± 0.013	< 0.001
Female sex	−0.40 ± 0.48	0.4	0.26 ± 0.23	0.3
Diabetes mellitus	−1.61 ± 0.73	0.028	NA^a^	—
Hydroxyurea use	−0.08 ± 0.47	0.9	0.06 ± 0.39	0.9
RAAS inhibitors use	−1.47 ± 0.58	0.011	NA^a^	—
Chronic RBC transfusions	0.57 ± 0.86	0.5	NA^b^	—
Tobacco smoking use	−1.36 ± 0.53	0.011	−1.03 ± 0.50	0.040
Body mass index (kg/m^2^)	0.002 ± 0.05	0.9	−0.042 ± 0.031	0.2
Systolic BP (mmHg)	−0.032 ± 0.02	0.049	−0.0004 ± 0.002	0.8
WBC count (× 10^3^/μL)	0.0078 ± 0.066	0.9	0.016 ± 0.036	0.7
Hemoglobin (g/dL)	0.45 ± 0.18	0.0095	0.11 ± 0.087	0.2
Platelet count (× 10^3^/μL)	0.0002 ± 0.001	0.9	0.0007 ± 0.001	0.4
Hemoglobin F (%)	0.037 ± 0.041	0.4	−0.029 ± 0.018	0.1
Absolute reticulocyte (× 10^3^/μL)	−0.00022 ± 0.0016	0.9	0.0016 ± 0.001	0.2
LDH (μ/L) (log‐transformed)	−0.69 ± 0.61	0.3	−0.81 ± 0.29	0.006
VOE Frequency (per year)	0.0035 ± 0.076	1.0	−0.055 ± 0.14	0.7
uACR (mg/g) (log‐transformed)	−0.70 ± 0.12	< 0.001	−0.32 ± 0.058	< 0.001
*APOL1* high‐risk	−2.11 ± 0.72	0.0035	−0.039 ± 0.49	0.9

*Note:* Beta‐values for LDH and Urine ACR are based on log‐transformed values.

Abbreviations: LDH, lactate dehydrogenase; RAAS, renin‐angiotensin‐aldosterone system; RBC, red blood cell; WBC, white blood cell; uACR, urine albumin‐to‐creatinine ratio; VOE, vaso‐occlusive episodes.

^a^ *N* = 1 for concurrent diabetes mellitus and for RAAS inhibitor use.

^b^ Chronic RBC transfusions were an exclusion criterion for entry in the Henri Mondor registry cohort.

In the Henri Mondor cohort, the median follow‐up was 8.2 years (interquartile range, 5.3–10.2 years) and the mean rate of eGFR decline was −1.50 mL/min/1.73m^2^ per year. On univariate analysis, baseline variables associated with a more rapid annual eGFR decline included older age, tobacco smoking, higher LDH and uACR concentrations (Table 2).

Next, we investigated the association between uACR and eGFR decline. In the UIC cohort, baseline uACR concentration as well as thresholds of ≥ 30, 100, and 300 mg/g creatinine were associated with a more rapid annual decline in eGFR after adjusting for age, sex, baseline eGFR, hydroxyurea and RAAS inhibitor use, and covariates with *p* ≤ 0.1 (Table 3). Using the uACR threshold of ≥ 100 mg/g creatinine, additional independent risk factors for a faster annual decline in eGFR included older age, hydroxyurea or RAAS inhibitor use, tobacco smoking, and higher baseline eGFR while a higher hemoglobin concentration was associated with a less rapid eGFR decline. Co‐inheritance of high‐risk *APOL1* status was independently associated with a faster eGFR decline (Table 4).

**TABLE 3:** Association of albuminuria with annual decline in estimated glomerular filtration.

UIC (*n* = 215)^a^				
Variable	eGFR change *β* ± standard error	*p*	eGFR decline ≥ 3 mL/min/1.73m^2^ OR, 95% CI	*p*
Baseline uACR (mg/g)^c^	−0.63 ± 0.13	< 0.001	1.54 (1.23–1.93)	< 0.001
Baseline uACR ≥ 30 mg/g	−1.49 ± 0.44	< 0.001	3.24 (1.47–7.14)	0.0036
Baseline uACR ≥ 100 mg/g	−1.74 ± 0.49	< 0.001	2.35 (1.12–4.94)	0.025
Baseline uACR ≥ 300 mg/g	−2.63 ± 0.57	< 0.001	2.69 (1.18–6.12)	0.019

Henri Mondor (*n* = 232)^b^				
Variable	eGFR change *β* ± standard error	*p*	eGFR decline ≥ 3 mL/min/1.73m^2^ OR, 95% CI	*p*
Baseline uACR (mg/g)^c^	−0.28 ± 0.073	< 0.001	1.61 (1.12–2.30)	0.009
Baseline uACR ≥ 30 mg/g	−0.55 ± 0.26	0.039	2.82 (0.68–11.64)	0.152
Baseline uACR ≥ 100 mg/g	−1.05 ± 0.28	< 0.001	4.81 (1.27–18.14)	0.021
Baseline uACR ≥ 300 mg/g	−1.92 ± 0.37	< 0.001	16.29 (3.56–74.57)	< 0.001

^a^ Adjusted for age, sex, baseline eGFR, hydroxyurea and RAAS inhibitor use, and covariates of *p* ≤ 0.1 (tobacco smoking, hemoglobin concentration, high‐risk *APOL1*).

^b^ Adjusted for age, sex, baseline eGFR, hydroxyurea use, and covariates of *p* ≤ 0.1 (tobacco smoking, baseline LDH (log)).

^c^ uACR values were log‐transformed.

**TABLE 4:** Covariates included in the multivariate model for the association of baseline uACR ≥ 100 mg/g with the rate of estimated glomerular filtration decline.

Variable	UIC (*n* = 215)		Henri Mondor (*n* = 232)	
	*β* ± standard error	*p*	*β* ± standard error	*p*
Age (years)	−0.068 ± 0.028	0.015	−0.056 ± 0.016	< 0.001
Female sex	−0.60 ± 0.45	0.18	0.32 ± 0.25	0.2
Hydroxyurea use	−0.93 ± 0.45	0.038	−0.25 ± 0.41	0.5
RAAS inhibitors use	−1.02 ± 0.56	0.070	—	—
Tobacco smoking use	−1.15 ± 0.48	0.019	−1.17 ± 0.47	0.013
Hemoglobin (g/dL)	0.43 ± 0.18	0.016	—	—
LDH (log‐transformed)	—	—	−0.66 ± 0.31	0.034
Baseline uACR ≥ 100 mg/g	−1.74 ± 0.49	< 0.001	−1.052 ± 0.28	< 0.001
Baseline eGFR	−0.033 ± 0.012	0.0049	0.034 ± 0.006	< 0.001
*APOL1* high‐risk	−1.63 ± 0.66	0.015	—	—

In the Henri Mondor cohort, baseline uACR concentration as well as thresholds of ≥ 30, 100, and 300 mg/g creatinine were associated with a more rapid annual decline in eGFR after adjusting for age, sex, baseline eGFR, hydroxyurea use, and covariates with *p* ≤ 0.1 (Table 3). Using the uACR threshold of ≥ 100 mg/g creatinine, additional risk factors for a faster annual decline in eGFR included older age, tobacco smoking, and higher LDH concentration while a higher baseline eGFR was associated with a less rapid eGFR decline (Table 4).

### 4.3 Rapid eGFR Decline > 3 mL/min/1.73m^2^

A rapid eGFR decline, defined as > 3 mL/min/1.73m^2^, was observed in 69 (26%) of the UIC cohort and 22 (7.4%) of the Henri Mondor cohort. Consistent with the rate of eGFR decline, a rapid decline in eGFR of > 3 mL/min/1.73m^2^ was independently associated with baseline uACR and uACR thresholds of ≥ 30, 100 and 300 mg/g in the UIC cohort and baseline uACR and uACR thresholds of ≥ 100 and 300 mg/g in the Henri Mondor cohort (Table 3).

Using a uACR threshold of ≥ 100 mg/g creatinine, other independent predictors for a rapid eGFR decline of > 3 mL/min/1.73m^2^ in the UIC cohort included tobacco smoking use (OR 3.08; 95% CI: 1.49–6.38; *p* = 0.0025) and older age (OR 1.05; 95% CI: 1.00–1.09; *p* = 0.029). In the Henri Mondor cohort, other independent predictors for a rapid eGFR decline included older age (OR 1.09; 95% CI: 1.02–1.17; *p* = 0.013) and tobacco smoking (OR 7.04; 95% CI: 1.08–45.7; *p* = 0.041).

## 5 Discussion

Kidney dysfunction is a common problem that consistently predicts increased morbidity and early mortality in patients with SCA. Clinical tools to identify patients at high risk for CKD progression as well as to guide lifestyle and disease modification to protect kidney function in this vulnerable population are urgently needed. In two independent cohorts of patients with Hb SS or Sβ^0^‐thalassemia from the United States and France, we demonstrate that the uACR and tobacco smoking are independent predictors of a more rapid decline in kidney function. Tobacco smoking promotes a pro‐inflammatory and oxidative milieu that leads to glomerulosclerosis, tubular atrophy, and mesangial proliferation [18]. Our findings underscore the importance of tobacco smoking cessation as part of routine medical care for those with SCA.

Albuminuria and proteinuria represent damage to the permselective barrier of the glomerular capillary wall to macromolecules [16]. While increased albuminuria is seen in the early stages of kidney disease and may propagate tubular damage leading to an acceleration of CKD progression in diabetic nephropathy, [11, 16] the ability of albuminuria and proteinuria to predict a rapid decline in kidney function in SCA is less clear. In one cohort of 175 SCA patients with a median follow‐up of 23 months, the rate of eGFR decline was significantly associated with baseline uACR (*r* = −0.37) in adults but not in children [10]. In another cohort of 290 SCA patients with a median follow‐up of 48 months, proteinuria estimated on dipstick urinalysis was not an independent predictor for a rapid eGFR decline [8]. Furthermore, there is also a lack of clarity on the baseline uACR threshold that represents an increased risk of rapid decline in kidney function. A baseline uACR of ≥ 100 mg/g creatinine has been proposed as a threshold to define persistent albuminuria and a greater risk of rapid eGFR decline in one study [10]. In another cohort of 535 SCA patients followed for a median of 5.3 years, uACR of 30–300 mg/g creatinine was not associated with progression to CKD stage II (HR 1.1; *p* = 0.8) while uACR > 300 mg/g creatinine was a risk factor for CKD progression (HR 3.9; *p* = 0.003) [19].

In our two independent cohorts with relatively long follow‐up periods, a higher uACR as either a linear variable or at a threshold of ≥ 100 mg/g creatinine independently predicted a more rapid decline in eGFR. Interestingly, a uACR threshold of ≥ 30 mg/g creatinine predicted a greater risk of eGFR decline ≥ 3 mL/min/1.73m^2^ in the UIC cohort but not in the Henri‐Mondor cohort. This discrepancy may be due to the variability in uACR at lower thresholds. Prior studies have demonstrated that only 49% of SCA patients with a uACR of 30–100 mg/g creatinine at baseline will have persistent albuminuria on repeat analysis [10]. Our data provide support for the American Society of Hematology guidelines that the uACR should be used as a screening tool for identifying patients with SCA at greater risk for a rapid decline in kidney function [12].

Limitations of our study include that both cohorts were observational studies which restrict our ability to investigate the effects of therapies, such as hydroxyurea or RAAS inhibitors, on changes in kidney function. There may have been other confounders between our two cohorts that affect the relationships of some variables, such as female sex and *APOL1* high‐risk status, with the rate of eGFR decline. Clinical differences, such as the prevalence of diabetes mellitus, hydroxyurea and RAASi use patterns, and the frequency of vaso‐occlusive episodes, may have contributed to the dissimilar relationships observed between our two centers. Unknown confounders, including diet, other genetic modifiers, and environmental exposures, may also be different between the two cohorts leading to discrepant findings. There is a potential for selection bias in our cohorts, representing patients with uACR values and > 3 months of follow‐up from two large comprehensive sickle cell centers. This may explain, in part, why the rates of eGFR decline in our analyses are not as severe as described in other cohorts of patients with SCA. Nevertheless, uACR was the most significant and consistent predictor for the rate of eGFR decline in both cohorts. The utility of uACR will need to be investigated in high SCA prevalence countries, such as Nigeria and India, to verify its predictive ability for kidney function decline in these settings.

In conclusion, we demonstrate that baseline uACR is a clinically important tool to predict kidney function decline in two independent and relatively large cohorts of Hb SS or Sβ^0^‐thalassemia patients with over 6 years of follow‐up. These results provide critical support for regular uACR screening as well as to identify patients for studies investigating the effects of SCA‐disease modifiers and for future clinical trials.

## Author Contributions

Study conception and design: P.B., E.A., V.A., F.G., G.L., C.B., C.A., O.E., J.H., M.A.R., J.P.L., V.R.G., S.L.S. Data collection, analysis and interpretation of results: P.B., E.A., V.A., F.G., G.L., C.B., J.H., M.A.R., J.P.L., V.R.G., S.L.S. Draft manuscript preparation: P.B., E.A., V.A., F.G., G.L., C.B., J.H., M.A.R., J.P.L., V.R.G., S.L.S. All authors critically reviewed and approved the final version of this manuscript.

## Funding

This work was supported by National Heart, Lung, and Blood Institute (R01 HL‐153161 and K24 HL‐177273); Novartis.

## Conflicts of Interest

P.B.: No relevant conflicts of interest (COI) related to this manuscript but has served as a consultant for Novartis, Addmedica, Bluebird Bio, Roche, Emmaus, Global Blood Therapeutics/Pfizer, Jazz Pharma and is an equity holder/co‐founder of INNOVHEM. V.A.: No relevant COI related to this manuscript but has received consulting fees from Addmedica, Vifor, Alnylam, and AstraZeneca. V.R.G.: No relevant COI related to this manuscript but has served as a consultant for Emmaus, Global Blood Therapeutics/Pfizer, and Modus Therapeutics and received research funding from Incyte and Novartis. S.L.S.: No relevant COI related to this manuscript but has served on advisory boards or as a consultant for Agios, BEAM therapeutics, Forma/Novo Nordisk, Novartis, Global Blood Therapeutics/Pfizer, Chiesi and Fulcrum. C.A. and O.E. are employees of Novartis Pharmaceuticals Corporation, USA. E.A., F.G., G.L., C.B., J.H., M.A.R., and J.P.L. declare no conflicts of interest.

## Data Availability

The data that support the findings of this study are available from the corresponding authors upon reasonable request.
